# Dysfunctional EAT thickness may promote maladaptive heart remodeling in CVD patients through the ST2-IL33 system, directly related to EPAC protein expression

**DOI:** 10.1038/s41598-019-46676-w

**Published:** 2019-07-17

**Authors:** Elena Vianello, Elena Dozio, Francesco Bandera, Gerd Schmitz, Manuela Nebuloni, Erika Longhi, Lorenza Tacchini, Marco Guazzi, Massimiliano Marco Corsi Romanelli

**Affiliations:** 1 Department of Biomedical Sciences for Health, University of Milan, Milan, Italy; 2 Cardiology University Department, Heart Failure Unit, IRCCS Policlinico San Donato, San Donato Milanese, Milano, Italy; 3 Department of Clinical Chemistry and Laboratory Medicine, University Hospital Regensburg, Regensburg, Germany; 4 U.O.C. of Surgical Pathology, Department of Biomedical and Clinical Sciences “Luigi Sacco”, University of Milan, Milan, Italy; 5 U.O.C. SMEL-1 of Clinical Pathology, IRCCS Policlinico San Donato, San Donato Milanese, Milano, Italy

**Keywords:** Mechanisms of disease, Cardiovascular diseases

## Abstract

Dysfunctional epicardial adipose tissue (EAT) secretome can influence the heart’s stretch response. However, the molecular mechanisms are still poorly understood. The aim of this study was to clarify how dysfunctional EAT promotes maladaptive heart remodeling in cardiovascular disease (CVD) through ST2 production associated with exchange protein directly activated by cAMP (EPAC) proteins. A series of 55 CVD males were enrolled and their EAT thickness, LV mass and volumes were measured by echocardiography. Blood, plasma and EAT biopsies were collected for molecular and proteomic assays. Taking EAT thickness as a continuous variable there was a direct correlation between the ST2 cardiac stretch mediator and EAT thickness (r = 0.54, p < 0.01) and an inverse relation between the ST2 gene and IL-33 expression (r −0.50, p < 0.01). In the CVD population EPAC2 expression directly correlated with the ST2 gene (r = 0.74, p < 0.0001) causing an ST2/IL-33 system local (p < 0.001) and systemic (sST2 = 57.33 ± 3.22 and IL-33 = 0.53 ± 017 pg/mL; p < 0.0001) protein imbalance associated with maladaptive remodeling. This indicated that dysfunctional EAT is a source of both EPAC and ST2 protein and an EPAC2 isoform seems involved in ST2 production in adipose tissue. Both EPAC2 and ST2 expression were directly related to maladaptive heart remodeling indices, suggesting EAT measurements could be useful in the early assessment of CVD complications.

## Introduction

Visceral adiposity is an important cardiovascular risk factor. As fat mass increases the fat cell secretome changes toward to a pro-inflammatory one which gives rise to a low-grade inflammatory state^1–4^, further promoting the onset and progression of cardiovascular diseases (CVD)^5–8^. Among the different types of visceral fat, epicardial adipose tissue (EAT) has drawn recent attention. The EAT mass has been associated with left ventricle (LV) mass remodeling, a common pathway of several CVD, including coronary artery disease (CAD) and valve dysfunctions^9–12^.

Among several processes potentially involved, recent studies have addressed ST2/IL-33 signaling as the main pathway promoting heart remodeling^13–15^ and only Gruzdeva *et al*. related this system to EAT thickness^16^. Stimulating growth factor 2 (ST2) is a member of the IL-1 receptor family of proteins and has two main isoforms: a transmembrane receptor (ST2L) and a soluble form (sST2)^15^. Under physiological conditions, myofibroblasts promote the release of the alarmin protein IL-33, the natural ligand of ST2L, which prevents hypertrophy, fibrosis and death of cardiac cells. When cardiac stretching occurs myofibroblasts and cardiomyocytes both release sST2 that sequesters IL-33 and blocks its binding to ST2L, promoting a compensatory response to the stretching^17,18^. Under pathological conditions, when there is detrimental biomechanical stretching the rise in sST2 production by cardiac cells further blocks IL-33 and leads to cardiac hypertrophy and fibrosis. sST2 is therefore now used as an additional tool for stratifying the risk of heart failure (HF)^13,14,17,19,20^.

There are recent reports that ST2 and IL-33 proteins are also produced by adipose tissue and are involved in the tissue homeostasis, with IL-33 working as an anti-inflammatory molecule that blocks adipocyte differentiation, lipid storage and M2 macrophage polarization^21–23^. However, the molecular mechanisms governing the expression of these proteins in adipose tissue are poorly understood. To our knowledge, there is only one study in a murine model demonstrating that the ST2/IL-33 system can be induced and controlled by the main ubiquitous second messenger in the body, cyclic adenosine 3′,5′-monophosphate (cAMP) through its more recently discovered effectors, called “exchange protein directly activated by cAMP” (EPAC)^24^. The EPAC protein family is composed of EPAC1 and EPAC2 which in adipose tissue control adipogenesis and lipolysis^25,26^. When the fat mass increases the up-regulation of cAMP-EPAC production activates downstream effectors which further promote adipose tissue expansion and inflammation^27–29^.

The present study had two main aims: (1) to assess whether the ST2/IL-33 cardiac stretch system could be a molecular link between the increase of EAT and maladaptive heart response in CVD patients; (2) to explore the role of EPAC proteins in the ST2-IL33 system in EAT because cAMP/EPAC signaling is pivotal in adipose tissue homeostasis.

## Results

### CVDs patient characteristics and classification

The 55 CVD male patients enrolled were stratified according to their heart pathology: 33 were CAD (ischemic) and 22 non-CAD (valvular). Anthropometric, clinical data and echocardiographic measurements related to heart remodeling are set out in Table 1. CAD patients had different degrees of vessel stenosis. Non-CAD cases had different kinds of valve stenosis or regurgitation. The CVD population had higher indices of body fatness than male reference values although they differed only in the HOMA index (p < 0.01) and HbA1c (p < 0.0001), indicating that the patients in both groups were overweight. EAT was thicker in CAD than non-CAD cases, although the difference was not significant.

**Table 1:** Demographic, clinical, anthropometric and echocardiographic characteritics of CVDs patients included in the study subdivided according to heart pathology.

	Valvular patients (non-CAD)	Ischemic patients (CAD)	*P*	male reference value
Age (years)	67,2 (52–80)	60,29 (41–81)	0.27	/
Systolic blood pressure (mmHg)	126,90 ± 10,32	123,60 ± 7,80	0.34	115–120
Diastolic blood pressure (mmHg)	71,54 ± 5,54	71,07 ± 4,97	0.78	75–80
Creatinine (mg/dl)	1,02 ± 0,25	1,01 ± 0,44	0.66	0,60–1,30
Fasting glucose (mg/dl)	88,14 ± 12,35	89,99 ± 15,19	0.89	60–99
HbA1c (%)	8,70 ± 15,02	5,63 ± 1,67	**0.0001**	<6,30
NT-PRO BNP (pg/ml)	736,10 ± 876,10	516,40 ± 929,60	0.42	<300
BMI	26,99 ± 4,10	26,85 ± 3,90	0.93	18,50–24,99
Weight (kg)	80,75 ± 17,79	75,31 ± 12,50	0.28	/
Height (m)	1,72 ± 0,08	1,67 ± 0,06	0.01	/
Waist (cm)	101,80 ± 17,24	101,30 ± 12,05	0.46	<94
Hip (cm)	105,70 ± 26,17	98,67 ± 13,00	0.18	/
WHR	0,98 ± 0,13	1,03 ± 0,08	0.17	<0,95
HOMA	1.47 ± 0.63	2.3 ± 2.00	0.01	<2,50
**Family history**				
hypertension	15	32	**0.005**	/
diabetes	6	15	0.2	/
CAD	13	16	0.77	/
**Echocardiographic data**				
EAT thickness in systole (mm)	6,90 ± 2,67	7,73 ± 2,11	0.26	^§^
**LV internal dimension**				
LV diastolic diameter (cm)	5,82 ± 1,05	5,20 ± 0,76	0.05	4,2–5,8
LV systolic diameter (cm)	3,79 ± 1,20	3,59 ± 0,84	0.77	2,5–4,0
**LV volumes (biplane)**				
LV EDV (mL)	156,10 ± 79,10	108,20 ± 44,13	**0.01**	62–150
LV ESV (mL)	69,01 ± 47,14	52,45 ± 36,32	0.18	21–61
**LV volumes normalized by BSA**				
LV EDV (mL/m^2^)	80,46 ± 35,36	58,19 ± 23,43	**0.006**	34–74
LV ESV (mL/m^2^)	34,62 ± 22,72	28,42 ± 20,09	0.13	11–31
LV EF function				
LV EF (%)	59,31 ± 10,25	54,61 ± 11,51	0.05	52–72
**LV mass by 2D method**				
septal wall thickness (cm)	1,28 ± 0,21	1,27 ± 0,88	**0.01**	0,60–1,00
RWT (%)	0,43 ± 0,12	0,42 ± 0,10	0.66	<42
LV mass (g)	334,80 ± 132,80	218,30 ± 61,12	**0.0002**	88–224
LV mass/BSA (g/m^2^)	163,30 ± 68.85	113,30 ± 36,81	**0.0009**	49–115
**LA size**				
LA (cm)	4,48 ± 1,04	3,90 ± 0,65	0.13	<4
**RV fucntion**				
TAPSE (mm)	107,60 ± 121,80	46,56 ± 65,08	0.88	>17
**Polmunary artery pressure**				
PAP (mmHg)	40,71 ± 12,38	28,08 ± 9,39	**0.01**	<35–40

Anthropometric, clinical and echocardiografic data of the cardiovascular patients, stratified according to their heart pathology as ischemic (CAD) or valvular (non-CAD). CAD patients had different degrees of vessel stenosis; Non-CAD had different kinds of valve stenosis or regurgitation. All had higher indices of body fatness than male reference values although they differed only in the HOMA index (p < 0.01) and HbA1c (p < 0.0001), indicating that both CAD and non-CAD groups were made up of overweight patients. EAT thickness was greater in CAD than non-CAD patients, but not significantly so.

Echocardiographic measurements of LV mass and volumes associated with heart remodeling were larger in non-CAD patients because of the pathogenesis of the valve disease and this was confirmed by higher NT-pro BNP levels in non-CAD than CAD cases (p < 0.0001). Both groups had preserved EF. The CAD patients were more likely to have a family history of hypertension.

Cardiovascular patients are stratified accoding to cardiovascular pathology in ischemic patients with different grade of aortic vessel stenosis and in valvular patients with different kind of valve disfunctions.

All data are expressed as mean ± standard deviation (SD).

All p less than 0.05 is considered statistically significative.

^§^The EAT thickness at level of right ventricle free wall is normally 7 mm in healthy lean individuals; no clinical cut off value is currently validated.

HbA1c, glycated hemoglobin; NT pro BNP, N-terminal prohormone of brain natriuretic peptide; BMI, body mass index; WHR, waist hip ratio; HOMA, homeostatic model assessment;

EAT, epicardial adipose tissue; LV, left ventricular; BSA, body surface area; EDV, end-diastolic volume; ESV, end-stystolic volume; EF, ejection fraction; RTW, relative wall thickness;

LA, left atrial; RV, right ventricle; TAPSE, tricuspid annular plane systolic excursion; PAP, pulmonary artery pressure.

LV mass and volumes associated with heart remodeling were higher in non-CAD, possibly because of the pathogenesis of valve disease, and this was confirmed by NT-pro BNP, which was also higher in non-CAD than CAD (p < 0.0001). All patients had preserved EF. CAD patients in particular tended more to have a family history of hypertension.

### BMI correlates with RTW in CVDs patients with eccentric hypertrophy

To understand better how body fat composition is involved with LV mass geometry changes, we divided our CVD population into four subgroups according to the kind of remodeling (Table 2): CVD patients with LV and normal geometry (RTW < 42%; LVM/BSA < 115 g/m^2^), concentric remodeling (RTW ≥ 0.42%; LVM/BSA < 115 g/m^2^), concentric hypertrophy (RTW ≥ 0.42%; LVM/BSA ≥ 115 g/m^2^) or eccentric hypertrophy (RTW < 0.42%; LVM/BSA ≥ 115 g/m^2^). The majority of CVD patients presented eccentric LV hypertrophy the first effect of chronic volume overload on LV, directly related to body fatness^30^. EAT thickness, considered a novel parameter of visceral adiposity, was lower in CVD patients with eccentric LV hypertrophy than the others, although not significantly (data not reported). Several studies acknowledge that EAT has an independent role in LV remodeling, especially since there is no reliable cut-off for normality^31^, so we used BMI as an independent variable to investigate how body fat composition was involved with LV changes. In patients with eccentric LV hypertrophy, the worst type of LV remodeling, BMI was positively related to RTW although the difference was only close to statistical significance (Spearman r = 0.40; p = 0.05).

**Table 2:** CVDs patients subdivided according to LV remodelling, LV hypertrophy associated to body fatness.

LV remodeling	Cardiovascular Patients (CVDs)	BMI	RTW (%)	LVM/BSA (g/m^2^)	EAT (mm)
Normal Geometry	13	26.84 ± 3.44	0.36 ± 0.05	90.69 ± 14.67	7.50 ± 2.20
Concentric Remodeling	2	27.65 ± 1.48	0.50 ± 0.06	106.50 ± 8.41	8.50 ± 0.70
Concentric Hypertrophy	18	25.86 ± 2.92	0.48 ± 0.08	155.80 ± 43.38	7.28 ± 2.64
Eccentric Hypertrophy	23	26.73 ± 3.80	0.37 ± 0.07	159.80 ± 56.44	6.95 ± 2.41
**Normal Geometry**					
**X BMI**	**RTW (%)**	**LVM/BSA (g/m**^**2**^**)**	**EAT (mm)**		
Spearman r	0.21	0.44	0.18		
p	0.45	0.13	0.55		
**Concentric Hypertrophy**					
**X BMI**	**RTW (%)**	**LVM/BSA (g/m**^**2**^**)**	**EAT (mm)**		
Spearman r	−0.08	0.11	0.21		
p	0.75	0.66	0.48		
**Eccentric Hypertrophy**					
**X BMI**	**RTW (%)**	**LVM/BSA (g/m**^**2**^**)**	**EAT (mm)**		
Spearman r	0.4	0.23	0.08		
p	**0.05**	0.29	0.74		

To clarify better how body fat composition and LV mass geometry changes were linked, we divided the CVD population into four groups on the basis of the kind of remodeling: CVD patients with LV with normal geometry (RTW < 42%; LVM/BSA < 115 g/m^2^), with concentric remodeling (RTW ≥ 0.42%; LVM/BSA <115 g/m^2^), with LV mass with concentric hypertrophy (RTW ≥ 0.42%; LVM/BSA ≥ 115 g/m^2^) and eccentric hypertrophy (RTW < 0.42%; LVM/BSA ≥ 115 g/m^2^). Correlational results indicated a linear correlation with RTW in CVD patients with eccentric LV hypertrophy as one of the main indices of maladaptive heart remodeling, although the difference was close to significance (p = 0.05).

BMI, body mass index; LVM/BSA, left ventricular mass on body surface area; RTW, relative wall thickness.

All p less than 0.05 is considered statistically significative.

### EAT increase correlates with ST2 and IL-33 molecular expression

**Figure 1:**
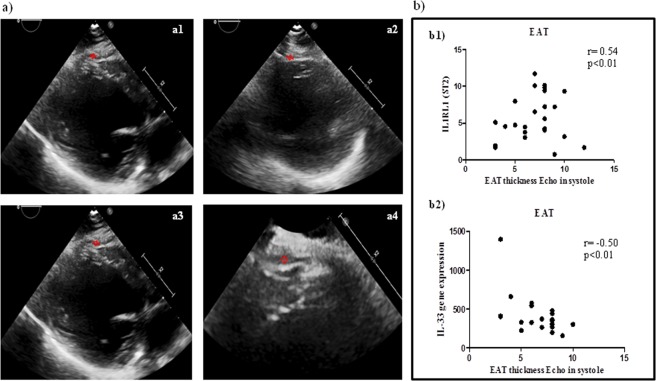
EAT thickness directly correlates with ST2 expression and inversely with IL-33. (**a**) End-diastolic (a1-a2) and end-systolic (b1-b2) echocardiographic frames, showing EAT thickness (red arrows). (**b**) Correlation results among EAT measurement and both ST2 molecular expression (r = 0.54, p < 0.0001) and IL-33 (Spearmann r = −0.50; p < 0.01) suggesting a potential involvement of fat body increase in ST2/IL-33 regulation.

EAT thickness were evaluated both in end-diastolic (Fig. 1a, a1-a2) and end-systolic echocardiographic (Fig. 1a, a3-a4) frames and we recorded and used as EAT measurement in end-systolic frame because is the better cardiac moment to appreciate EAT thickness as previously described^31,32^.

To circumvent the lack of a recognized clinical cut-off for EAT thickness, we took the EAT measurement as a continuous variable to assess the effect of its increase. Considering the role of ST2/IL-33 signaling as a cardiac stress response system, we ran a correlation analysis among the EAT thickness, ST2 and IL-33 expression in EAT biopsies from our CVD population (Fig. 1b). There was a linear correlation between EAT and the ST2 molecular expression (r = 0.54, p < 0.0001) and IL-33 (Spearman r −0.50; p < 0.01), suggesting that body fat mass was indeed involved in ST2/IL-33 regulation.

### ST2/IL-33 expression in EAT

In view of the role of ST2/IL-33 signaling in promoting maladaptive heart remodeling, we investigated the gene expression and protein synthesis of ST2 and IL-33 in EAT.

**Figure 2:**
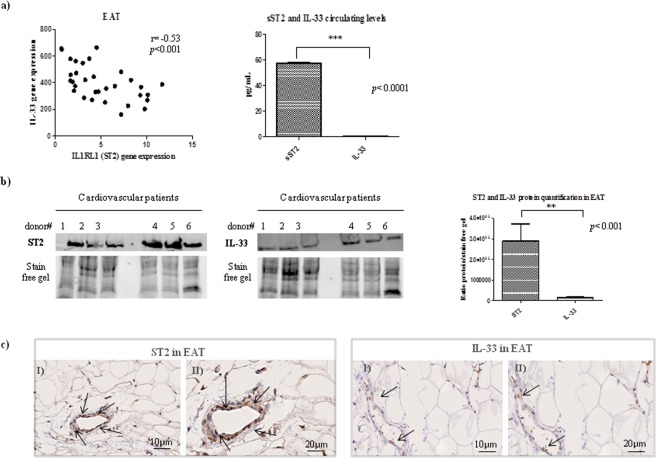
ST2 and IL-33 cardiac stretch mediators expression, production and immunolocalization in EAT. Under biomechanical stretchING the main cytokines involved in compensatory remodeling in the heart are ST2 and IL-33. We measured their expression, protein production and total circulating levels in EAT biospies from CVD patients. (**a**) EAT presents significantly higher expression of IL-33 gene than IL1RL1 (ST2), and ST2 and IL-33 expression is inversely correlated. sST2 total circulating level is higher than Il-33 circulating protein. (**b**) Western blot shows that EAT is a source of the ST2 cardiac stretch mediator and IL-33 cardioprotective proteins. (**c**) Representative images of EAT biopsies with immunoreaction for ST2 and IL-33 positive cells in separate panels (panels II with magnification). Both ST2 immunoreactivity and IL-33 are present in EAT biopsies specially those close to endothelial vessels.

Figure 2a shows the correlation results between the IL1RL1 (as ST2) and IL-33 genes, presented in arbitrary units and our date shown an inverse correlation between ST2 gene expression and IL-33 (Spearman r = 0.53, p < 0.001).

Regarding the protein production of the ST2/IL-33 system, our CVD population presented higher systemic (2a) and local protein levels of ST2 than IL-33 alarmin (Fig. 2b), confirming the molecular control of ST2 on IL-33 production both systemically and in EAT tissue of CVD patients.

The ST2/IL-33 immunolocalization in EAT biopsies (Fig. 2c) was mainly around the endothelial vessels, ST2^+^ and IL-33^+^ cells, confirming the activation of the ST2/IL-33 immune system in EAT, suggesting their involvement in adipose tissue metabolism during an increase in fat mass.

### EAT thickness is associated to increased expression of EPAC1 and EPAC2 proteins

**Figure 3:**
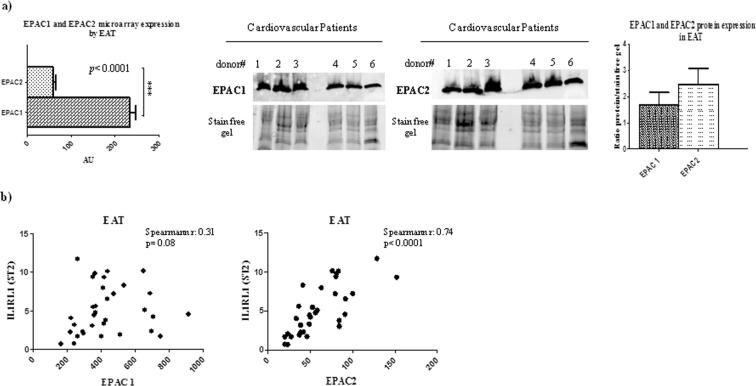
Adipose tissue size regulators EPAC1 and EPAC2 are express in EAT and EPAC2 directly correlates with IL1RL1 expression. EPAC proteins, the main effectors of cAMP as controllers of adipose tissue size and metabolism, are expressed by EAT from CVD patients. (**a**) EPAC1 isoform is significantly overexpressed compared to the EPAC2 isoform in EAT cells, as shown by microarray results (p < 0.0001) but the total protein production, analyzed by Western blot and quantified normalizing the data using stain-free gels, indicated higher expression of EPAC2 protein than EPAC1, although the difference was not significant. (**b**) Correlation analysis between the expression of EPAC1 and EPAC2 adipose tissue size controllers and the ST2 cardiac stretch mediator in dysfunctional EAT biopsies. Only the EPAC2 isoform directly correlates with the ST2 gene level (Spearman r = 0.74, p < 0.0001). No differences were found between the EPAC1 and ST2 gene.

Because EPACs are the main controllers of adipose tissue size as effectors of cAMP, we quantified EPAC1 and EPAC2 expression in EAT at the gene and protein levels (Fig. 3). The EPAC1 gene level was higher than EPAC2 in EAT from CVD biopsies (p < 0.0001), but protein production seemed higher in the EPAC2 isoform than EPAC1, although not significantly so (Fig. 3a).

Since ST2 was induced by cAMP/EPAC signaling in a murine model, we made a correlation analysis at gene level of EAT biopsies. There was a linear correlation between ST2 and EPAC2 (r = 0.82, p < 0.002) (Fig. 3b) but no correlation with EPAC1 (Fig. 3b), or between EPAC isoforms and IL-33 (data not shown).

### ST2 expression in EAT is associated to maladaptive heart remodeling

Because ST2 is the main cardiac stretch mediator associated with maladaptive heart remodeling and excessive body mass, we observed higher ST2 levels were higher and clinical signs of heart remodeling, so we explored the potential association of EAT molecular patterns with heart remodeling through ST2/IL-33 signaling. CVD patients were classified in two groups on the basis of the median ST2 gene level and correlation studies were done to compare the genes and clinical features. CVD patients with lower ST2 expression levels (Table 3) presented a negative correlation of ST2 with IL-33, EAT thickness and sST2. sST2 levels too were negatively correlated with EAT thickness. Table 4 shows the correlations in patients with higher ST2 expression. EAT thickness positively correlated with RTW (r = 0.59, p < 0.05) and inversely with IL-33. ST2 correlated positively with EPAC2 and IL-33 inversely with EAT thickness, cardiac indices of remodeling (LVM, hLVM, EDPW, RTW and diastolic pressure) and total circulating IL-33. IL-33 circulating levels correlated directly with some indices of remodeling (diastolic pressure, LVM, hLVM and PAP) and inversely with EPAC2. EPAC2 directly correlated with ST2 and inversely with total circulating IL-33.

**Table 3:** Correlation pattern of EAT biopsies expressing minest ST2 gene.

Correlation pattern of EAT biopsies expressing miner IL1RL1 (<12.72 AU)			
X	Y	Spearman r	p
EAT thickness measurament	sST2 total circlating level (pg/ml)	−0.68	**0.04**
IL1RL1 (ST2) gene expression	NT-pro BNP (pg/ml)	−0.88	**0.001**
	IL-33 gene expression (AU)	−0.69	**0.006**
	sST2 total circlating level (pg/ml)	−0.54	**0.04**
sST2 total circlatin levels	EAT thickness measurament (mm)	−0.68	**0.04**
	IL1RL1(ST2) gene expression (AU)	−0.54	**0.04**
IL-33 gene expression	IL1RL1 (ST2) gene expression (AU)	−0.69	**0.006**

Correlation patterns of EAT expressing lower levels of ST2 gene than the median (12.72 AU). CVD patients expressing low levels of the ST2 cardiac stretch mediator presented a cardioprotective profile against maladaptive remodeling. Spearman correlation analysis was used for relations among different variables.

**Table 4:** Correlation pattern of EAT biopsies expressing highest ST2 gene.

Correlation pattern of EAT biopsies expressing highest IL1RL1 (≥12.72 AU)			
X	Y	Spearman r	p
EAT thickness measurament	RTW (%)	0.59	**0.03**
	IL-33 gene expression (AU)	−0.52	0.05
IL1RL1 (ST2) gene expression	EPAC 2 (AU)	0.55	**0.03**
IL-33 gene expression	EAT thickness (mm)	−0.52	0.05
	hLVM (%)	−0.65	**0.01**
	LVM (g)	−0.64	**0.01**
	EDPW (cm)	−0.66	**0.008**
	RTW (%)	−0.62	**0.01**
	Diastolic pression (mmHg)	−0.75	**0.007**
	IL-33 toatal circulating level (pg/ml)	−0.69	**0.01**
IL-33 total circulating level	Diastolic pression (mmHg)	0.66	**0.03**
	Fasting glucose (mg/dl)	0.67	**0.01**
	LV diastolic diameter (cm)	0.53	0.05
	LVM (g)	0.66	**0.01**
	hLVM (%)	0.75	**0.002**
	PAP (mmHg)	0.84	**0.002**
	EPAC 2 (AU)	−0.71	**0.008**
EPAC 2 gene expression	IL1RL1 (ST2) gene expression (AU)	0.67	**0.01**
	IL-33 total circulating level (pg/ml)	−0.65	**0.03**

Correlation patterns of EAT expressing higher levels of ST2 gene than the median (12.72 AU). Compared to CVD patients with lower ST2 expression patients expressing high levels of the ST2 cardiac stretch mediator presented a pro-maladaptive remodeling profile. Spearman correlation analysis was used for relations among different variables.

### CVD patients with high sST2 plasma levels had an EAT-derived alarm molecular pattern associated with maladaptive heart remodeling

Since sST2 acts as a decoy receptor silencing ST2/IL-33 cardioprotective signaling against maladaptive remodeling, we divided CVD patients according to the median sST2 circulating level (57.42 pg/mL) to verify any relationship between sST2 levels and EAT amount. Table 5 reports correlation analyses in CVD patients with the lowest sST2 levels (<57.42 pg/mL). There was a direct relation between ST2 and the EPAC2 gene and an inverse relation with remodeling indices (LV diastolic diameter, LV EDV and hLVM). Total circulating IL-33 directly correlated with the remodeling indices and inversely with the EPAC2 gene. EPAC2 gene expression negatively correlated with the remodeling indices and total circulating IL-33 and positively with the ST2 gene.

**Table 5:** Correlation pattern of EAT biopsies from CVDs patients with minest sST2 circulating protein (<57.42 pg/ml).

Correlation pattern of EAT biopsies from CVDs patients with minest sST2 circulating protein (<57,42pg/ml)			
X	Y	Spearman r	p
IL1RL1 (ST2) gene expression	EPAC 2 (AU)	0.73	**0.007**
	LV diatolic diameter (cm)	−0.68	**0.006**
	LV EDV (ml/m^2^)	−0.83	**0.003**
	hLVM (g/m^2^)	−0.58	**0.02**
IL-33 total circulating level	Diastolic pression (mmHg)	0.59	**0.01**
	LV diatolic diameter (cm)	0.47	**0.02**
	EF (%)	0.42	**0.04**
	h LVM (g/m^2^)	0.43	**0.03**
	EPAC2 gene expression (AU)	−0.59	**0.02**
EPAC2 gene expression	LV EDV (ml/m^2^)	−0.66	**0.01**
	h LVM (g/m^2^)	−0.54	**0.04**
	IL1RL1 gene expression (AU)	0.57	**0.03**
	IL-33 total circulating level (pg/ml)	−0.59	**0.02**

Correlation patterns of CVD patients expressing lower sST2 total circulating levels than the median for the total CVD population (57.42 pg/mL). The CVD patients releasing a low level of sST2 protein into the circulation presented a cardioprotective profile against maladaptive remodeling. Spearman correlation analysis was used for relations among different variables.

Correlation analyses in CVD patients with the highest sST2 levels (>57.42 pg/mL) gave a positive correlation between ST2 and EPAC2 and with IL-33 gene expression. This latter related positively with circulating sST2 and inversely with ST2 (Table 6).

**Table 6:** Correlation pattern of EAT biopsies from CVDs patients with highets sST2 (>57.42 pg/ml).

Correlation pattern of EAT biopsies from CVDs patients with highets sST2 (>57,42 pg/ml)			
X	Y	Spearman r	p
IL1RL1 (ST2) gene expression	EPAC 2 (AU)	0.73	**0.007**
sST2 total circulating level	NT-pro BNP (pg/ml)	0.65	**0.002**
	HbA1 (%)	0.37	**0.04**
	IL-33 expression gene (AU)	0.7	**0.008**
IL-33 gene expression	sST2 total circulating levels (pg/ml)	0.69	**0.008**
	IL1RL1 expression gene (AU)	−0.51	0.05
EPAC2 gene expression	ST2 expression gene (AU)	0.66	**0.01**

Correlation patterns of CVD patients expressing higher sST2 total circulating levels than the median of the total CVD population (57.42 pg/mL). CVD population releasing high levels of sST2 protein into the circulation presented a correlation pattern associated with maladaptive remodeling. Spearman correlation analysis was used for relations among different variables.

## Discussion

The present study looked translationally at the potential role of EAT in LV remodeling in CVD. We measured the amount of EAT using a simple non-invasive method based on echocardiographic measurements of adipose tissue thickness. Though there is no definite consensus on the most effective threshold for prognosis stratification, we used EAT measurement as a continuous variable to gain a better picture of how the body fat increase is involved in LV remodeling.

Lacking a clinical cut-off for EAT we assessed the effects of a body fat increase on LV changes using BMI, as suggested by Iacobelli *et al*.^33^ to investigate how fat mass influences heart rearrangement. The majority of our CVD patients presented eccentric LV hypertrophy which is the main kind of LV remodeling associated with chronic volume overload in case of obesity. Our CVD population of overweight individuals was also stratified according to heart pathology as CAD or non-CAD.

As regards the impact of BMI on heart remodeling, there was a linear association between BMI and RTW only in patients with eccentric LV hypertrophy, suggesting a relationship between geometrical remodeling and metabolic activity mediated by adipose tissue. Although we did not find any linear correlation between EAT and BMI, both being indices of visceral adiposity, we can justify this on the basis that EAT can change size depending on the severity of heart disease, due to the presence of necrotic and apoptotic areas after immunoinfiltration in the tissue^34^.

Considering the potential link between the extent of EAT and the maladaptive cardiac remodeling, we investigated the molecular pattern of EAT and its mechanistic role. With the particular anatomical position of this visceral deposit, several studies have demonstrated its vasocrine and paracrine control of subtending tissues including the heart. We found that in comparison to patients with physiological EAT thickness, the dysfunctional EAT related to the greater amount of tissue expressed and produced unbalanced levels of the ST2/IL-33 system, with detrimental cardiac metabolism and responses.

ST2 and IL-33 are expressed and well-characterized in the cardiovascular system since they control cardiac responses to biomechanical stretching in case of hemodynamic overload. During biomechanical stress, cardiac fibroblasts produce IL-33 alarmin protein, the natural ligand of ST2 receptor, and this binding promotes anti-fibrotic and anti-inflammatory intracellular signaling in cardiomyocytes. However, in CVD the soluble form of ST2 derived from alternative splicing of the ILRL1 gene acts as a decoy receptor, blocking ST2L/IL-33 binding, and promotes pro-fibrotic and pro-inflammatory signaling leading to maladaptive remodeling.

The mechanisms underlying the effect of EAT mass increase and ST2 production have been investigated little. Our molecular and proteomic findings indicated that dysfunctional EAT can be a source of ST2 for neighboring cardiac cells in CVD patients, with significant increases of both local and circulating ST2. In contrast, in dysfunctional EAT the expression and production of IL-33 alarmin protein seemed to be reduced, losing its local and systemic protective effects. Therefore the central role of the amount of EAT in maladaptive remodeling is further supported by immunohistochemical results showing both IL-33 and ST2 positive cells, especially close to endothelial vessels, suggesting they shift through the microcirculation.

There appear to be no reports as yet of any molecular pathway associated with fat mass increase and ST2 expression and production. Because chronic changes in adipose tissue morphology and metabolism were counterbalanced by β3 receptor activation, with a consequent increase in intracellular cAMP^26^ and a murine study suggested the induction of ST2/IL-33 by cAMP effectors^24^, the present investigation, for the first time, looked into the possible relation between EPAC production by dysfunctional EAT and ST2 expression. Our data suggested that abnormally thick EAT expressed and locally produced EPAC1 and EPAC2 proteins, and the EPAC2 isoform positively correlated with the expression of ST2 without any association with IL-33 alarmin expression; this suggests a link between increased EAT mass and ST2 expression.

To gain a better understanding of the EPAC and ST2/IL-33 axis in cardiovascular outcomes, we investigated the molecular patterns of EAT biopsies driven by ST2 expression, dividing patients according to their median ST2 expression. CVD patients with the lowest ST2 expression had a protective molecular pattern related to cardiovascular remodeling, with an inverse relation between ST2 and local IL-33 alarmin gene, EAT thickness and the total circulating sST2 cardiac stretch mediator. EAT size was also inversely related with total circulating sST2 protein, suggesting a pivotal role of EAT metabolism in ST2 expression. CVD patients with the highest ST2 expression, on the other hand, had an unfavorable molecular pattern associated with geometrical remodeling, indicative of a direct relation between EAT thickness and RTW and an inverse relation with alarmin IL-33 gene expression. In this group of patients the protective role of IL-33 gene on cardiac stretching seemed to be lost, due to the inverse correlations among the main indices of remodeling, including RTW, and total circulating IL-33 alarmin protein with EPAC2 gene expression. EPAC2 expression directly correlated with the expression of the ST2 cardiac stretch mediator, suggesting a possible regulatory implication in ST2/IL-33 signaling.

To gain more knowledge of the pivotal role of increased EAT on the ST2 induction associated with maladaptive remodeling, we divided CVD patients according to the median sST2 level as the main circulating mediator associated with the maladaptive heart response. CVD patients with low release of sST2 presented a protective profile associated with heart remodeling, with inverse relations between IL1RL1 and EPAC2 gene expression and LVM, hLVM, and LV EDV cardiac indices. IL-33 total circulating protein as alarmin directly correlated with indices of heart enlargement, to counterbalance the effects of stretching on myofibroblasts. IL-33 circulating protein was inversely related with EPAC2 expression as a potential new inducer of ST2 in EAT. However, CVD patients with high release of sST2 presented an alarmin profile associated with maladaptive remodeling in which the sST2 cardiac stretch mediator correlated inversely with local expression of IL-33 gene in EAT; ST2 maintained the positive relation with EPAC2 expression, suggesting the potential role of this cAMP effector in ST2 expression in adipose tissue.

In summary, our findings point to an unknown role of the amount of EAT in maladaptive cardiac remodeling in CVD patients with preserved EF, without signs or symptoms of HF. In dysfunctional EAT, EPAC2 and ST2 expression seems to act antagonistically on IL-33 cardioprotective protein production. The consequent imbalance of the ST2/IL-33 system can have direct effects on maladaptive heart remodeling, suggesting its involvement in CVD complications.

## Methods

### Study population and ethics

This study enrolled 55 CVD male patients at the IRCCS Policlinico San Donato (San Donato Milanese, Milan, Italy) scheduled for open heart surgery. Patients with recent acute myocardial infarction, malignant disease, prior major abdominal surgery, renal failure, end-stage heart failure (HF) and more than 3% change in body weight in the previous three months were excluded. Demographic, anthropometric and clinical data including age, sex, and family history of hypertension, diabetes and CAD were recorded. As indicated by the preoperative coronary angiographic findings, 33 were ischemic patients with coronary artery disease (CAD), undergoing elective coronary artery bypass grafting surgery (7 CAD2 and 26 CAD3, with different grades of stenosis) and 22 were non-CAD patients undergoing valve replacement (12 aortic stenosis, 7 aortic insufficiency and 3 mitral insufficiency). Before surgery, EAT thickness was measured by echocardiography. CAD and non-CAD patients were then stratified on the basis of a median EAT thickness of 8 mm as an indicator of maladaptive adipose tissue accumulation.

The study protocol was approved by the local ethics committee (ASL Milano Due, protocol no. 2516) and patients gave written informed consent to the examination protocol, conducted in accordance with the Declaration of Helsinki, as revised in 2013.

### Blood collection and measurements

Blood samples were collected after overnight fasting into pyrogen-free tubes with ethylenediaminetetraacetic acid as anticoagulant. Plasma samples were separated after centrifugation at 1000 g for 15 min and were stored at −20 °C until analysis. Fasting glucose, glycated hemoglobin (HbA1c), creatinine and N-terminal pro B-type natriuretic peptide (NT-pro BNP) were quantified with commercial kits using a Cobas 6000 analyzer (Roche Diagnostics, Milan, Italy). sST2 and IL-33 in plasma were measured by enzyme-linked immunosorbent assays (ELISA) (R&D Systems, Minneapolis, MN, USA).

### Quantification of EAT and collection

EAT was quantified before surgery by echocardiography with a 2.5- to 3.5-MHz transducer probe (Vingmed-System Five; General Electric, Horten, Norway). EAT thickness was measured along the free wall of the right ventricle from the parasternal long- and short-axis views and is generally identified as the echo-free space between the outer wall of the myocardium and the visceral layer of the pericardium; it is analyzed perpendicularly on the free wall of the right ventricle at end-systole, as reported^31^. This point is where EAT generally is the thickest and is measurable more easily^31^. EAT thickness at the right ventricle free wall ranges from 1 mm to almost 23 mm, but is normally about 7 mm in healthy lean males and females; no clinical cut-off is currently validated on account of the differences in size in different ethnic groups^31^.

EAT biopsy samples were harvested adjacent to the proximal right coronary artery prior to starting cardiopulmonary bypass pumping. For gene expression analysis, biopsies were stored in Allprotect Tissue Reagent (Qiagen, Hilden, Germany) at −20 °C until RNA and protein extraction. For immunohistochemical staining, biopsies were immediately fixed in 4% paraformaldehyde.

### Echocardiography data of left ventricular mass (LV)

Pre-surgical resting echocardiography (Vingmed-System Five; General Electric, Horten, Norway) was done to examine systolic, diastolic and valvular morphology and function. LV hypertrophy was defined according to current guidelines for echocardiographic chamber quantification^11^.

The measures were LV diastolic diameter (reference values (RV): male 4.2–5.8 cm), LV systolic diameter (RV: male 2.5–4.0 cm), LV end-diastolic volume (EDV) (RV: male 62–150 mL), LV end-systolic volume (ESV) (RV: male 21–61 mL), LV ejection fraction (EF) (RV: male 52–75%), septal wall thickness (RV: male 0.6–1.0 cm), relative wall thickness (RWT) (RV: male <0.42%), LVM (RV: male 88–224 g), indexed LV mass (h LVM) (RV: male 49–115 g/m^2^), left atrium (LA) (RV: male <4 cm), tricuspid annular plane systolic excursion (TAPSE) (RV: male >17 mm), pulmonary artery pressure (PAP) (RV: male <35–40 mmHg).

### Microarray expression analysis

Total RNA was extracted from EAT biopsies with the RNeasy Lipid Tissue Kit (Qiagen). RNA concentration was quantified by NanoDrop 2000 (ThermoScientific, Wilmington, DE) and RNA integrity was checked using the Agilent RNA 6000 Nano kit and the Agilent 2100 Bioanalyzer (Agilent Technologies, Santa Clara, CA). Gene expression was analyzed with a one-color microarray platform (Agilent): 50 ng of total RNA was labeled with Cy3 using the Agilent LowInput Quick-Amp Labeling kit-1 color. RNA was purified with the RNeasy Mini Kit (Qiagen) and the amount and labeling efficiency were measured with NanoDrop. Hybridization was done using an Agilent Gene Expression Hybridisation Kit, scanning with an Agilent G2565CA Microarray Scanner System. Data were processed using Agilent Feature Extraction Software (10.7) with the single-color gene expression protocol, and raw data were analyzed with ChipInspector Software (Genomatix, Munich, Germany). In brief, raw data were normalized on a single-probe level based on the array mean intensities and statistics were calculated using the SAM algorithm by Tusher. Gene expression of RAPGEF3 (encoding for EPAC1), RAPGEF4 (encoding for EPAC2), remodeling mediators including IL1RL1 (encoding for ST2) and IL-33 were expressed in arbitrary units (AU).

### Western blot analysis

EAT tissue was homogenized in RIPA buffer containing 50 mM Tris-HCl, pH 7.5, 150 mM NaCl, 1 mM EDTA, 1 mM EGTA, 1% Triton X-100, 0.1% SDS, 0.5% Na-deoxycholate, 50 mM sodium fluoride plus 1% protease inhibitor cocktail (Sigma-Aldrich, MO), in Tissue Lyser II (Qiagen). Aliquots of 50 μg of total proteins were electrophoresed on SDS Mini-PROTEAN^®^ TGX^TM^ Stain-Free Precast Gels (BIO RAD) and transferred to nitrocellulose membranes of the Trans-Blot^®^ Transfer System Pack (BIO RAD) on a Trans Blot^®^ Turbo^TM^ device (BIO RAD). Membranes were first blocked with 5% non-fat dry milk/TBS with 0.1% (vol/vol) Tween 20 for 1 h, then incubated overnight at 4 °C with primary antibodies for: EPAC1 (Cell Signaling, dilution 1:1000), EPAC2 (Cell Signaling, dilution 1:1000), ST2 (Proteintech, dilution 1:500) and IL-33 (Proteintech, dilution 1:700). After washing, membranes were incubated with horseradish peroxidase (HRP)-labeled secondary antibodies for 2 h at room temperature. Immunoreactive protein bands were then detected using an ECL chemiluminescence kit (BIO RAD) using the ChemiDoc MP Imaging System (BIO RAD). Densitometric analyses were done with Image Lab 5.2.1 software (BIO RAD). Data were normalized to the total protein after stain-free blot, and presented as the percentage density/volume (%). All Western blots were run at least in triplicate for each experimental group.

### ST2 and IL-33 immunohistochemical staining in EAT sections

Deparaffinised EAT sections were rehydrated and antigen was retrieved by autoclaving in sodium citrate buffer 0.01 M pH 6 for 5 minutes at 120 °C. After rinsing in PBS 1X, endogenous peroxidase activity was quenched in 0.3% H_2_O_2_ in PBS for 20 minutes. To block unspecific binding, sections were incubated with normal swine serum (Dako Cytomation) and then with the following primary antibodies: mouse monoclonal anti-human ILRL1 (diluted 1:400 in PBS, Proteintech) and mouse monoclonal anti-human IL-33 (diluted 1:200 in PBS, Proteintech), both overnight. Sections were rinsed in PBS and processed for amplification of the immune signal with anti-mouse HRP-polymer complex (MACH 1 Universal HRP-Polymer detection, Biocare Medical). Biocare’s Betazoid DAB was used to develop color. Sections were counterstained with Mayer’s hematoxylin and mounted with Mowiol 4–88.

Immunohistochemical reactions were observed with a Nikon Eclipse 80i microscope and images were acquired with a digital camera and the image acquisition software.

### Statistical analysis

Data were expressed as mean ± standard (SD) and analyzed by GraphPad Prism 5.0 biochemical statistical package (GraphPad Software, Inc., San Diego, CA). The normality of data distribution was assessed by the Kolmogrov-Smirnoff test. Comparison between groups was performed using two-tailed unpaired Student t test or Mann-Whitney U-test as appropriate. Spearman or Pearson correlation analysis were used to examine association among different variables. All differences with *p* < 0.05 was considered statistically significant.

The microarray analysis was performed using Agilent Genespring 12.6 which transform raw data in log2 and baseline-transformed to median of all sample. Each individual gene was analyzed with Welch’s t-test and the p value result from the correction with Benjamini-Hochberg FDR.

In order to better understand the involvement of ST2 expression by EAT in heart metabolism and remodeling, we performed correlation analysis using two different cut off: (1) the median value of ST2 molecular expression (12.72 AU); (2) the median value of sST2 circulating level (57.42 pg/ml) to determine any differences among EAT expression pattern and indexes of LV remodeling.

### Ethics approval and consent to participate

The Local Ethics Committee ASL MILANO DUE approved this study (Protocol No. 2516). All patients provided informed consent.
