# Author Correction: Dysfunctional EAT thickness may promote maladaptive heart remodeling in CVD patients through the ST2-IL33 system, directly related to EPAC protein expression

**DOI:** 10.1038/s41598-020-58143-y

**Published:** 2020-01-21

**Authors:** Elena Vianello, Elena Dozio, Francesco Bandera, Gerd Schmitz, Manuela Nebuloni, Erika Longhi, Lorenza Tacchini, Marco Guazzi, Massimiliano Marco Corsi Romanelli

**Affiliations:** 1 Department of Biomedical Sciences for Health, University of Milan, Milan, Italy; 2 Cardiology University Department, Heart Failure Unit, IRCCS Policlinico San Donato, San Donato Milanese, Milano, Italy; 3 Department of Clinical Chemistry and Laboratory Medicine, University Hospital Regensburg, Regensburg, Germany; 4 U.O.C. of Surgical Pathology, Department of Biomedical and Clinical Sciences “Luigi Sacco”, University of Milan, Milan, Italy; 5 U.O.C. SMEL-1 of Clinical Pathology, IRCCS Policlinico San Donato, San Donato Milanese, Milano, Italy

Correction to: *Scientific Reports* 10.1038/s41598-019-46676-w, published online 17 July 2019

This Article contains errors. In Figure 1a, the incorrect image was used for panel a3, and the labelling of these panels was inconsistent. The correct Figure and Legend appears below, in Fig. 1.

**Figure 1:**
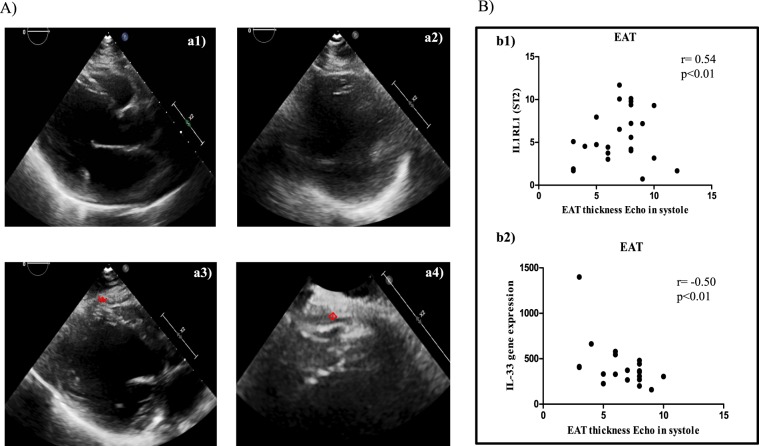
EAT thickness directly correlates with ST2 expression and inversely with IL-33. (**A**) Echocardiographic frames showing EAT thickness (red arrows): a1, long axis end-diastolic frame; a2, short axis end-diastolic frame; a3, long axis end-systolic frame; a4, zoom of long axis view of end-systolic. (**B**) Correlation results among EAT measurement and both ST2 molecular expression (r = 0.54, p < 0.0001) and IL-33 (Spearmann r = −0.50; p < 0.01) suggesting a potential involvement of fat body increase in ST2/IL-33 regulation.

The scale bars provided in Figure 2c are incorrect. The correct Figure 2c appears below as Fig. 2, with information on magnification provided in the legend.

**Figure 2:**
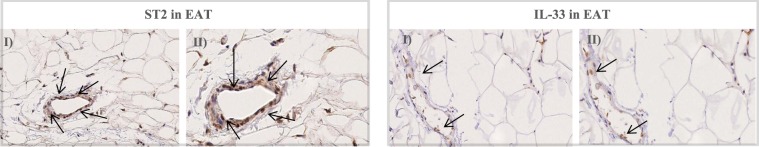
Representative images of EAT biopsies with immunoreaction for ST2 and IL-33 positive cells in separate panels (panel **I** at 10X and panel **II** at 20X of magnification respectively). Both ST2 immunoreactivity and IL-33 are present in EAT biopsies specially those close to endothelial vessels.

The Supplementary Information did not include full-length gel images. Uncropped images for gels used in Figure 2b and 3b are provided below as Fig. 3.

**Figure 3:**
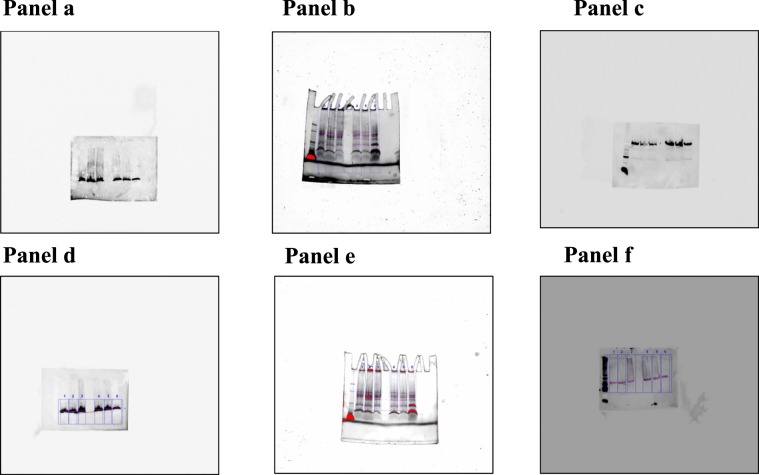
**(Panel a)** shows the full gel provided in Figure 3a of the Article as EPAC1; **(Panel b)** shows the stain-free gel shown in Figures 2b and 3a of the Article, and used as normalizer for ST2 and EPAC1 immunoblots after stripping phase; **(Panel c)** shows the full membrane provided in Figure 2b of the Article as ST2; **(Panel d)** shows the full membrane provided in Figure 3a of the Article as EPAC2; **(Panel e)** shows the gel for a stain free gel shown in membrane provided in Figures and 2b and 3a of the Article, and used as normalizer for IL-33 and EPAC2 immunoblots after stripping phase; **(Panel f)** shows the full gel provided in Figure 2b of the Article as IL-33.

